# Sleeping under the Ocean: Despite Total Isolation, Nuclear Submariners Maintain Their Sleep and Wake Patterns throughout Their Under Sea Mission

**DOI:** 10.1371/journal.pone.0126721

**Published:** 2015-05-27

**Authors:** Marion Trousselard, Damien Leger, Pascal van Beers, Olivier Coste, Arnaud Vicard, Julien Pontis, Sylvain-Nicolas Crosnier, Mounir Chennaoui

**Affiliations:** 1 Institut de recherche biomédicale des armées (IRBA), Unité Stress, Brétigny-sur-Orge, France; 2 Université Paris Descartes, Sorbonne Paris Cité, APHP, Hôtel-Dieu, Centre du sommeil et de la vigilance, Paris, France; 3 Université Paris Descartes, Sorbonne Paris Cité, Equipe d'accueil VIgilance FAtigue SOMmeil (VIFASOM) et santé publique EA 7330, Paris, France; 4 Institut de recherche biomédicale des armées (IRBA), Unité Fatigue et Vigilance, Brétigny-sur-Orge, France; 5 HIA Desgenettes, Unité sommeil, Lyon, France; 6 Escadrille des Sous marins lanceurs d’engins (SNLE), Service médical de l’ESNLE, Brest, France; Kent State University, UNITED STATES

## Abstract

**Background:**

To assess the effects of isolation, inadequate exposure to light and specific shift work on the subjective and objective measurements of sleep and alertness of submariners.

**Purpose:**

A strictly controlled randomized crossover study with the polysomnography recorded twice during the mission.

**Methods:**

**Setting:** Shift and night work with prolonged (70 days) social isolation from the real world (with no phone or Internet contact with families or friends during a routine mission aboard the “Téméraire” French Strategic Submarine with Ballistic Nuclear missiles (SSBN). **Participants:** 19 submariners working on a 24-hour shift for three days in a row schedule. **Interventions:** The participants attended two polysomnographic (PSG) recordings of night sleep on Day 21 (D21) and Day 51 (D51) of the 70-day patrol; urine cortisol levels were also taken after sleep, and subjective assessments of sleep, sleepiness, mood and anxiety on D21 and D51. The light and temperature on board were also recorded.

**Results:**

PSG analyses showed that sleep did not significantly vary in length (total sleep time) or in quality between D21 and D51. The mariners reported the same subjective sleep, sleepiness, anxiety or mood (except for a slightly worse score for confusion on D51). Blood cortisol levels did not vary significantly.

**Conclusions:**

These results show that humans living in an isolated environment for more than two months with this specific shift schedule do not suffer from any significant effects on sleep, sleepiness and confusion between D21 and D51, when they follow an organized regular shift pattern with controlled light and temperature.

## Introduction

Although the United Nations considers that the vast majority of countries around the world have lived in peace since the beginning of the century, six countries still have a military presence in the oceans, using nuclear-powered strategic submarines (NPSS) to deter new conflicts [1–2]. Among NPSS, around 30 Submersible, Ballistic, Nuclear submarines (SSBN) have specific strategic missions with nuclear missiles at board and serve as undetectable launch platforms for intercontinental missiles in case of nuclear conflict. These SSBNs belong to the United States (14), Russia (6), France (4), the United Kingdom (4), the People's Republic of China, and India [2]. During long-term missions, these submarines are in total isolation from the real world, with no normal means for the crew to communicate except by secrecy codes. In addition to the advanced technology involved, the security of these submarines, and of world peace, depends on various human factors that include the sleep and wake patterns of crews exposed to long periods of shift and night work and to prolonged (70 days) social isolation. With no exposure to external light, submariners could have non-entrained sleep and wake patterns, which may affect their ability to react promptly. i.e. It has been shown that American submariners did not follow an imposed 18-h-cycle [3]. A compressed-work schedule (ALT), built to enhance circadian rhythm entrainment and sleep hygiene, was tested on subjective sleep in 40 American submariners [4], with cortisol and temperature measurements in a sub-sample of 10 subjects. The ALT submarine schedule was a close 6, 3-section watch-system with 6-hours-on-6-hours-off-6-hours-on, 12-hours-off, 6-hours-on-6-hours-off-6-hours-on, 24-hours-off. This schedule was reminiscent of the close 4 schedule designed by Kleitman and including 24 hours of on-watch time per 72 hours [5]. However sleep was discovered to be shorter with this new system, which was therefore not deemed as efficient compared to the classic 18 hour watch system [4].

It has indeed been clearly shown that social isolation and the lack of regulation by the light-dark solar cycle exposes humans and animals to a desynchronization of their biological clock, with irregular sleep/wake patterns and disturbance of other circadian rhythms, such as melatonin and cortisol secretion and internal temperature regulation [6–10]. These observations have been described in blind people who have no light perception [11–13], but also in subjects only exposed to dim light in laboratories and without sufficient exposure to light in professional settings [14–15]. Both groups of subjects complained of sleep disorders, which robustly reflects the lack of biological clock training [10–14]. Shift work alone and its relative lack of light exposure is well-known for its ability to disturb the biological clock and sleep/wake patterns [16–21] and to potentially alter wake and cognition processes [22–24]. Major negative impacts of shift-work are disturbed, shortened sleep and impaired vigilance, well characterized by the 3^rd^ international classification of sleep disorders (ICSD-3) [25], and defined as shift-work disorders. Poor sleep concerns about 50% of shift workers, insomnia 30% and impaired alertness 25%, with possible important consequences in specific settings, for example, oil rig workers in the North Sea or astronauts in the Mars 520-d mission simulation [26–27].

Therefore, the goal of the survey was to compare sleep and alertness of our submariners’ group at two separate times during the mission in order to capture the evolution of sleep in isolated and shift conditions.

## Methods

### Setting

**Fig 1:**
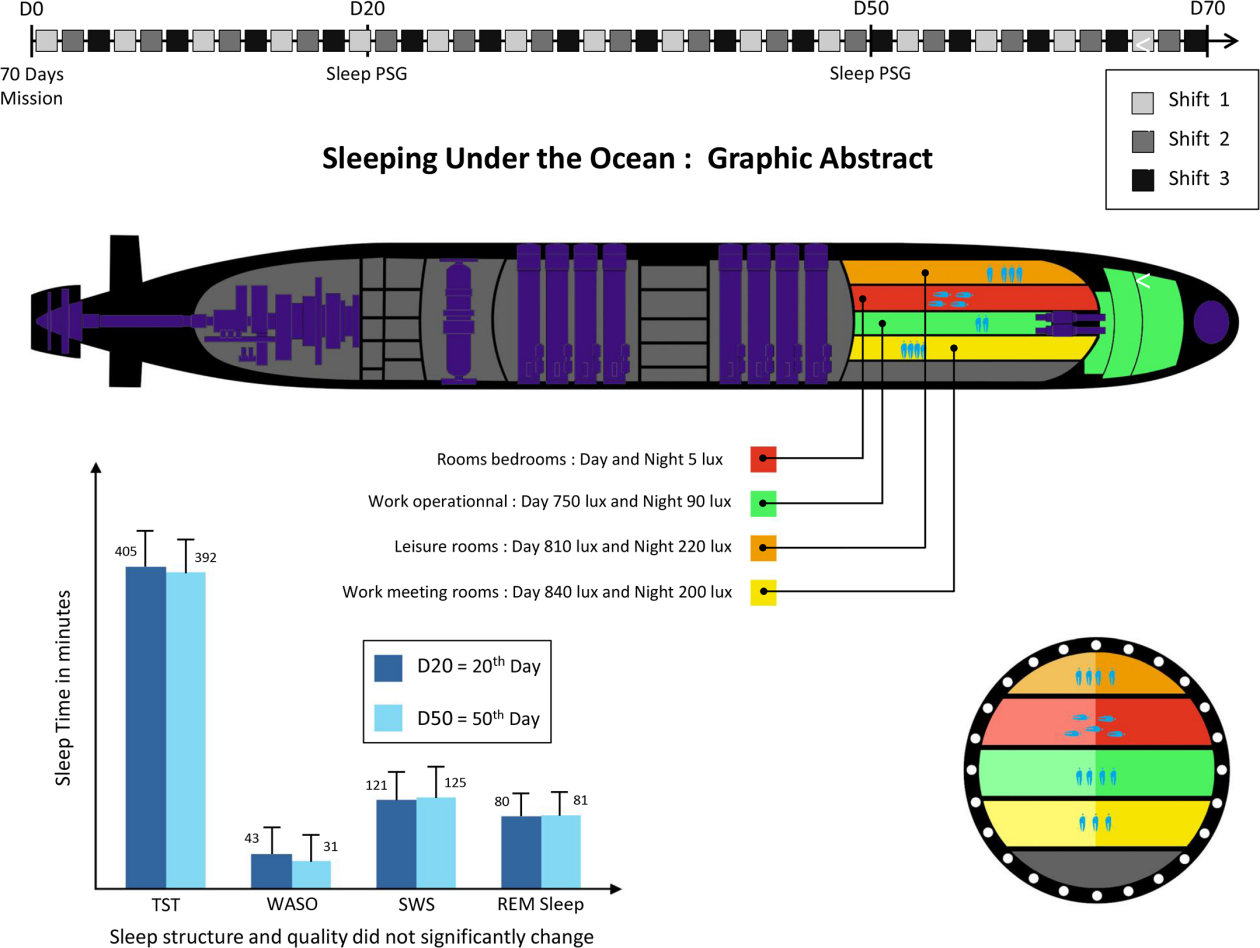
The top of the "Fig. 1" shows the 70-day long mission with the 3-day shift schedules to better understand at what point of the mission the polysomnography (PSG) recordings were performed. The submarine “Téméraire” is also shown schematically with the different levels of lighting in the “work”, “leisure” or “sleep” rooms. The graph at the bottom left of the "Fig. 1" shows the evolution of objective sleep parameters at the two points during the mission. Durations of TST (total sleep time), WASO (Wake after sleep onset), SWS (slow wave sleep), and REM (Rapid Eye movement) sleep did not vary significantly.

The study was performed during a 70-day mission on board the SSBN French submarine “Le Téméraire” in 2011. The environmental conditions inside the SSBN were strictly controlled "Fig 1".

Light was scheduled to reproduce a 24-hour daylight/nighttime cycle, with 16 hours of daytime levels (90 to 840 lux) and 8 hours (from midnight to 8 a.m.) with night lighting (mostly red and varying from 5 to 220 lux). We measured the luminosity levels for the submariners included in the study both at eye-level in the horizontal plane of vision and in the direction of the light source to assess night and day levels. Temperature was maintained between 18 and 25°C (64 to 77°F). For security reasons, noise was also specifically controlled because it is crucial that submarines are undetectable to other boats. Nuclear-based turbine propulsion and electromagnetic suspension of turbines make the SSBN submarines very quiet and it is said that it is even “more silent than the sea itself” [28].

Throughout the mission, the crew was totally isolated from the “real world”: no phones, no Internet, no external light, and no link with families or friends outside the submarine.

### Subjects

The protocol, approved by the French Direction Générale de l’Armement (DGA)occupational safety and health ethics committee (DGA CHSCT), was explained in detail to the 100 male crew members, one month before departure and the first 20 volunteers (mean age 28 years, SD 7.22, range 19–46) signed an informed consent and were retained for inclusion in the study. The experimental protocol conformed to the international ethical standards [29].

The criteria for exclusion were any presence of one or several chronic diseases, or any treatment affecting sleep or vigilance. But, due to Navy safety regulations, subjects with non-controlled chronic medical or psychiatric diseases are prohibited from participating in long-term under sea missions. Confidentiality of the results was guaranteed by the anonymous treatment of the data.

### The shift schedules during the 70-day patrol "Fig 2"

**Fig 2:**
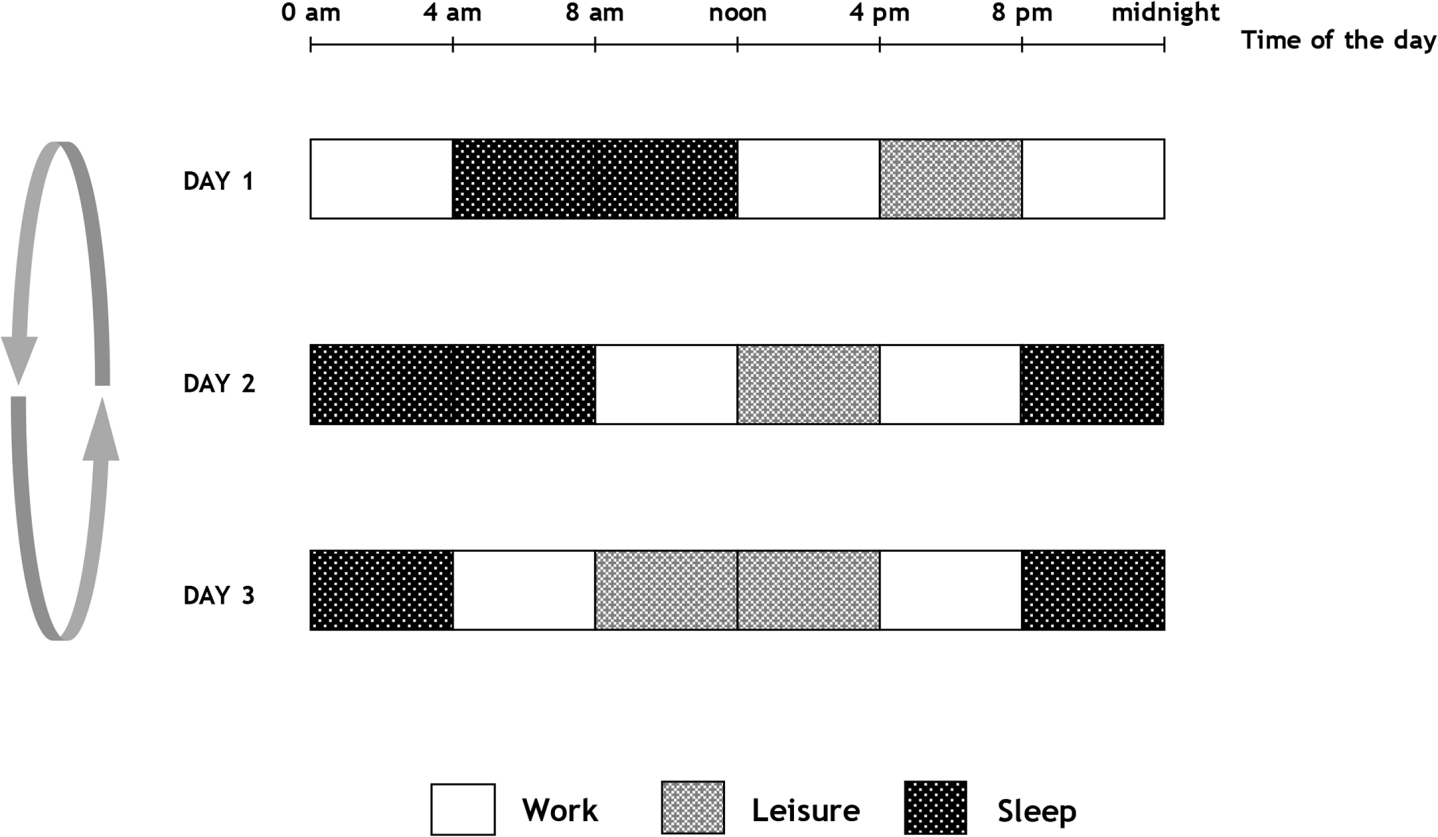
Schedules of submariners on a 3 days cycle.

**-** The crew was divided into three teams and submariners worked throughout the mission on a 3-day shift rotation (see Fig 2). Operational work demanded high concentration and was limited to a 4 consecutive hours shift system. Each work period was followed by at least 4 hours of leisure time or sleep. During leisure time, submariners mainly shared common activities, including meals, training, cleaning and security exercises. They also had short periods of personal time to read, listen to music, practice physical activity or watch videos.

Sleep was scheduled in three 8-hour periods and one 4-hour additional period. During the sleep periods, crew members were instructed to go to their rooms and encouraged to get as much sleep as possible.

- The goal of the survey was to compare sleep and alertness at two separate times during the mission in order to capture the evolution of sleep in isolated and shift conditions. All parameters were collected twice, at the same time during each shift. The first night’s recording was performed on the second day of the shift, between midnight and 8 am, the 21^st^ day of the mission (D21) 19 days i.e., 7 shifts, after the first D2. We realized that when we recorded on the second day of the shift "Fig 2" we maximized the opportunity of having a better sleep. A second assessment was performed on day 51 (D51), 10 shifts later. We decided not to conduct any baseline assessment before boarding, because the nights before boarding were often shortened and disturbed by a heavy work load and anxiety linked to the coming familial and social separation on such prolonged isolated missions [30].

### Behavioural sleep, alertness and mood: subjective assessments

Subjective ease of getting to sleep and sleep restoration were assessed using the Buguet sleep scale, based on visual analogue scales (VAS) previously used on male members of the army and in reference to the previous night [31]. Sleepiness was assessed using the Epworth sleepiness scale (ESS), in reference to the month prior to the assessment: a score of 10 or more indicated that the subject was sleepy; a score of 16 or more indicated being very sleepy [32]. Anxiety and depression were estimated on the French short-version of the Profile of Mood Scale (POMS) [33]. The POMS consists of an adjective checklist of 37 items rated on a five-point scale that ranges from (1) “not at all” to (5) “extremely”. Subjects were asked to answer according to their present mood. Six dimensions were then calculated: anxiety-tension, depression-dejection, anger-hostility, fatigue-inertia, vigor-activity and confusion-bewilderment. These subjective assessments were noted the same day of each objective polysomnography.

### Sleep polysomnography (PSG) recording and analyses

On-board ambulatory PSG was performed using a miniature digital device (Actiwave, Camtech ltd. UK) during the participants’ normal sleep time. Standard set-up and criteria for scoring sleep stages were used [34]. Arousals, leg movements and respiratory events were scored according to guidelines provided by the American Academy of Sleep Medicine [35]. Recordings were only made during the night and the submariners had to stay in bed for 8 hours from light extinction at midnight to morning awakening at 8 am. According to AASM sleep analysis rules [35], the files were analyzed by two experienced sleep technologists to calculate the total sleep period (TSP) in minutes (min), total sleep time (TST, min), sleep onset latency (SOL, min), sleep efficiency 1(SE1, percentage) which was calculated as a ratio between TST/TSP, and sleep efficiency 2 as a ration between TST/TIB (Time in bed, which was imposed to 8 hours) and the wake after sleep onset (WASO, min). The percentages of sleep stages (Stage 1 and 2 = light sleep; Stage 3 = Slow wave sleep (SWS); Rapid Eye Movement (REM) sleep and REM sleep latency) were also assessed. Finally, for the SWS and REM periods, the following parameters were retained during each night recorded: number of periods, average period (min), shortest period (min), longest period (min), and average intervals between SWS periods and REM periods (min).

### Cortisol level assessments

12 h nighttime urine cortisol excretion was assessed (overnight + 2 hours before and 2 hours after) on D21 and D51. Cortisol concentrations were measured using radioimmunoassay kits based on the protein concentration rates (Siemens Healthcare Diagnostics, Germany). The urinary cortisol excretion rates were calculated based on diuresis and the creatinine excretion rates. All sample analyses were double-checked.

### Statistical analyses

All data were treated as ordinal except for subjective items. For socio-demographic, sleep and psychological variables, the 19 submariner samples were considered. The evolution of sleep and psychological parameters during the mission were assessed using student “paired t tests” comparison tests between session-points (D21 and D51). Correlations between variables were assessed by Spearman rho correlation analyses. All comparisons between groups were carried out using non-parametric tests due to the group size differences and to non-normative distribution of some set of data (particularly for subjective data). All analyses were performed using SPSS 17.0 for Windows (SPSS GmbH Software, Munich). Data are expressed as mean and standard deviation (SD). All statistical tests were two-tailed. P< 0.05 was considered as statistically significant.

## Results

Twenty male volunteers (mean age 28 years, SD 7.22, ranging 19 to 46 years old, 13 were between 19 and 29, 5 between 30 and 40, and 2 above 40) were selected to take part in the study. For one subject, the second sleep assessment was not completed so we analyzed the results from 19 subjects. The participants had served an average of 9.8 years (SD 4.65) on active duty and an average of 4.4 years (SD 3.22) on SSBN patrols. Eight of the participants (42.1%) were married.

### Sleep behaviour, alertness and mood: subjective assessments (Table 1)

Subjectively, the crew did not report a significant difference in the quality of sleep or ease of falling asleep between D21 and D51. There was no significant difference in sleep onset latency (SOL) or number of awakenings between the two nights. Sleepiness assessed by the ESS was normal at the first assessment and slightly >10 at the second, but these differences were not statistically significant.

**Table 1:** Subjective and objective sleep variables, sleepiness and mood at D21 and D51.

	D21		D51		D51 vs. D21, p
Variables	Mean	SD	Mean	SD	
**Subjective sleep**					
Quality of sleep VAS	4.07	2.73	3.78	3.05	0.31 (0.76)
Ease of falling asleep VAS	6.75	2.95	6.67	2.89	0.08 (0.93)
SOL (min)	33.05	25.73	35.89	33.31	-0.28 (0.77)
Number of awakenings	2.73	1.36	2.42	2	0.56 (0.57)
**Sleepiness (ESS)**	8.89	3.43	10.63	2.73	-1.72 (0.09)
**PSG Sleep characteristics**					
SOL (min)	10.82	12.54	11.71	8.53	-0.24 (0.8)
TSP (min)	448.76	81.37	423.88	67.34	2.02 (0.06)]
TST (min)	405.61	69.47	392.65	55.6	0.99 (0.32)
WASO (min)	43.16	39.80	31.24	20.37	1.11 (0.27)
SE1 (TST/TSP) %	0.89	0.08	0.91	0.04	-0.74 (0.46)
SE2 (TST/Time in bed = 8 hours) %	0.84	0.14	0.88	0.14	-0.65 (0.39)
% REM	0.20	0.04	0.22	0.05	-1.02 (0.31)
REM latency (min)	81.31	14.25	77.21	15.2	0.67(0.25)
% NSW	0.46	0.05	0.41	0.08	2.66 (0.02)
%SWS	0.30	0.05	0.33	0.09	-1.94 (0.06)
**Cortisol (nmol/L)**	100.42	58.68	91.38	62.34	0.46 (0.64)
**Mood and anxiety (POMS)**					
Anxiety tension	2.36	2.92	3.84	4.72	-1.15 (0.22)
Vigour	14.78	7.77	12.31	4.07	1.23 (0.22)
Fatigue	2.15	1.89	3.57	2.91	-1.78 (0.08)
Depression	0.63	1.11	2.26	3.82	-1.79 (0.08)
Anger	0.94	2.09	1.68	2.54	-0.97 (0.33)
Confusion	0.89	1.66	2.31	2.35	**-2.14 (0.03)**

Subjective and objective sleep variables, sleepiness, cortisol, mood and anxiety were assessed on the 21st day of the mission, D21, and 30 days later (after 10 shifts) at D51 = the 51st day. Values were compared using student “t” comparison tests between session-points. No significant differences were observed in subjective values of the quality of sleep and ease of falling asleep assessed on visual analogue scales (VAS) or sleep onset latencies (SOL) expressed in minutes (min) or on the number of awakenings. There was also no statistical difference in sleepiness over the last month on the Epworth sleepiness scale [11]. Objective sleep values measured by polysomnography (PSG) were also not significantly different between D21 and D51, including: SOL, total sleep period (TSP), total sleep time (TST), wake after sleep onset (WASO), sleep efficiency 1 (SE1) = TST/TSP, sleep efficiency 2 = TST/time in Bed (TIB), the percentages (%) of rapid eye movement (REM) sleep and slow wave sleep (SWS) and the REM latency. No significant difference was observed in the 12-hour night-cortisol excretion assessed on the same days. Mood and anxiety were assessed based on the profile of mood state (POMS) six dimensions [12] and no significant differences were observed except for confusion, which was significantly higher on D51. **Normal range values:** Subjective values: Sleepiness was assessed using the Epworth sleepiness scale (ESS), referring to the month prior to the assessment: a score of 10 or more indicated that the subject was sleepy; a score of 16 or more indicated being very sleepy (12). Objective values: in a young and healthy group of subjects normal SOL is under 30 minutes, common total sleep time in adults is around 420 minutes, normal REM latency is above 60 minutes, normal % of REM sleep is above 20% and % of SWS is above 20%. Normal WASO in less than 60 minutes, normal SE is above 90% (Ohayon et al., 2004).

Analyses of POMS scores showed that the degree of confusion increased between D21 and D51 but there was no change in feelings of fatigue, depression, anxiety or vigour.

### Sleep polysomnography (PSG) recording and analyses (Table 1 *)*

Objectively, total sleep time (TST) did not change significantly during the mission, with an average of 405.6 minutes (SD 69.5) at D21, which was short but within the normal range, and 392.6 minutes (SD 55.6) at D51 (p = 0.99). The TSP reflects compliance with sufficient time spent in bed: subjects were asked to stay in bed for 8 hours and they turned off the lights for 448.8 minutes (SD 81.4) at D21 and 423.9 minutes (SD 67.4) at D51. This slight decrease in TSP may be explained by the slight but not statistically significant increase in SE1 (91% [SD 4%] at D51 vs. 89% [SD 8%] at D21, p = 0.74).

SOL, WASO and the percentages of SWS, REM, and NSWS did not vary significantly from D21 to D51. REM latency was also not significantly different from D21 to D51. SE2 (TST/TIB) reflects a lower efficiency 84% at D21, but not significantly increased at 89% at D51.

There were no significant differences in the blood cortisol assessed at the end of the night with an average 100.4 nmol/l (SD 58.7) at D21 and 91.4 nmol/l (SD 62.3) at D51 (p = 0.46).

### Correlations between subjective data, objective sleep data and blood cortisol data

Correlation analyses applied to objective sleep variables on D21 showed that the submariners with greater sleep efficiency 1 (TST/TSP) had shorter SOL (r^2^ = -0.93; p<0.001), shorter WASO (r^2^ = -0.98; p<0.001), a higher percentage of NSWS (r2 = 0.62; p = 0.02), and a tendency towards a lower percentage of SWS (r^2^ = -0.47; p = 0.10) than those with lower sleep efficiency 1.

Correlation analyses, applied to objective sleep variables on D51, showed that the submariners with greater sleep efficiency had shorter WASO (r^2^ = -0.86; p<0.001), and a tendency towards a lower percentage of REM (r^2^ = -0.51; p = 0.058) and a higher percentage of SWS (r^2^ = 0.52; p = 0.07) than those with lower sleep efficiency.

Submariners with the highest cortisol urinary excretion exhibited a tendency towards a lower sleep efficiency (r^2^ = -0.49; p = 0.10), a longer WASO (r^2^ = 0.52; p = 0.07), and a higher percentage of REM (r^2^ = 0.53; p = 0.08). At D51 submariners with the highest cortisol urinary excretion had the highest percentage of REM (r^2^ = 0.61; p = 0.026), and a tendency towards a higher STP (r^2^ = 0.46; p = 0.1) and to a lower percentage of SWS (r^2^ = -0.47; p = 0.1).

The correlation analyses between objective sleep variables and psychological subjective variables (Buguet sleep Scale and POMS) at D21 are presented on Table 2. Results showed that submariners with higher confusion levels had lower sleep efficiency (r^2^ = -0.72; p<0.01), longer SOL (r^2^ = 0.72; p<0.01) and longer WASO (r^2^ = 0.74; p<0.01). Other significant associations have also been underlined on Table 1A.

**Table 2:** Correlation between objective polysomnographic results and subjective (Buguet and POMS) on D21.

	Quality of sleep	Ease of falling asleep	SOL Sub.	Number of awakenings	POMS Anxiety Tension	POMS Vigour	POMS Fatigue	POMS Depression	POMS Anger	POMS Confusion
SOL	0.31 p = 0.21	-0.39 p = .11	**0.49 p = .04**	0.04 p = .87	0.367 p = .905	.1067 p = .729	-.2527 p = .405	-.2016 p = .509	-.1443 p = .638	**-.72 p<0.01**
TSP	**0.52 p = .027**	-0.16 p = .52	0.17 p = .49	0.42 p = .09	.1098 p = .721	**.4951 p = .085**	-.2287 p = .452	-.0469 p = .879	-.2031 p = .506	-.3106 p = .302
TST	0.31 p = .21	-0.02 p = .94	-0.05 p = .85	0.35 p = .15	-.3321 P = .268	.1964 P = .520	**-.5436 P = .055**	-.1790 P = .558	**.5080 P = .76**	**-.5242 P = .066**
WASO	**0.51 p = .03**	-0.3 p = .23	0.44 p = .07	0.23 p = .36	-.0235 p = .939	-.5840 p = .36	-.3028 p = .315	-.0758 p = .806	.1016 p = .741	**.74 p<0.01**
SE	-0.39 p = .11	0.30 p = .23	-0.42 p = .08	-0.12 p = .65	-0.048 p = .988	**.5588 p = .047**	-.3020 p = .316	.0757 p = .806	-.1548 p = .614	**0.72 p<0.01**
%REM	**0.63 p = .005**	-0.20 p = .43	**0.46 p = .05**	0.39 p = .11	-.1345 p = .661	-.4315 p = .141	.1266 p = .680	.0240 p = .938	.2976 p = .323	.1716 p = .575
%SWS	-0.22 p = .38	-0.11 p = .68	0.04 p = .86	-0.26 p = .29	-2962 p = .326	-.2114 p = .488	.1228 p = .689	-.2616 p = .388	-.3733 p = .209	-.2056 p = .500

The same analyses at D51 are presented on Table 3. Results showed that the submariners with longer TST also had a significantly better feeling on their SOL and sleep quality. Moreover those who reported the highest number of nocturnal awakenings exhibited a lower percentage of sleep in SWS (r^2^ = -0.71; p = 0.01) and of REM sleep (r^2^ = -0.99; p = 0.002).

**Table 3:** Correlation between objective polysomnographic results and subjective (Buguet and POMS) on D51.

	Quality of sleep	Ease of falling asleep	SOL Sub.	Number of awakenings	POMS Anxiety Tension	POMS Vigour	POMS Fatigue	POMS Depression	POMS Anger	POMS Confusion
SOL	-0.21 p = .40	0.042 p = .873	-0.29 p = .27	-0.21 p = .40	-.4482 p = .125	-.1902 p = .534	-.1014 p = .742	-.2960 p = .326	-.4565 p = .117	-.1530 p = .618
TSP	-0.42 p = .09	0.396 p = .12	-0.45 p = .07	-0.001 p = .99	.1253 p = .532	.3656 p = .247	-.1478 p = .714	-.0588 p = .758	-.2252 p = .652	-.3325 p = .421
TST	**-0.49 p = .04**	0.45 p = .06	**-0.49 p = .04**	-0.14 p = .59	.1098 p = .721	.4554 p = .118	-.0047 p = .988	-.0752 p = .807	-.2546 p = .401	-.1764 p = .564
WASO	-0.03 p = .92	0.07 p = .77	-0.14 p = .59	0.37 p = .14	-.1464 p = .633	-.0260 p = .933	-.3492 p = .242	-.1249 p = .684	-.3571 p = .231	.3965 p = .180
SE	0.03 p = .91	0.03 p = .92	0.17 p = .51	-0.24 p = .35	0.2475 p = .415	.1033 p = .737	.3821 p = .198	.1910 p = .532	.4552 p = .118	-.3637 p = .222
%REM	-0.12 p = .66	0.01 p = .96	-0.15 p = .57	**.99 0.002**	-.2172 p = .476	.2027 p = .507	.1173 p = .703	-.1074 p = .727	.2388 p = .432	-.1405 p = .647
%SWS	0.30 p = .24	-0.07 p = .80	0.07 p = .78	**.71 p = 0.01**	.1240 p = .687	-.4848 p = .093	.3001 p = .319	;1861 p = .543	.0281 p = .927	.5207 p = .068

These tables present correlations between subjective assessments of sleep (Buguet sleep Scale (Buguet et al., 1981)and POMS (Profile of Mood Scale; Shacham, 1983) and objective measurements by polysomnography on the 21^st^ day (D21) and the 51st day (D51) of the mission. SOL = Sleep Onset latency; TSP = Total sleep period; TST = Total Sleep Time; WASO = Wake after sleep onset; SE = sleep Efficiency 1; %REM = percentage of Rapid Eye Movement sleep; %SWS: percentage of slow wave sleep; **significant correlations are bolded.**

## Discussion

When we designed this study to observe and record the sleep patterns of this submariners’ crew exposed to shift work and an absence of light exposure through their long mission under the ocean, we hypothesized that we would find significant sleep and wake complaints and sleep reduction and disturbances [19–20,23–24]. Very few studies have previously observed sleep/wake patterns of submariners [3–5]. In the first survey carried out in nuclear submarines, the melatonin rhythms of 20 American submariners with an imposed 18-hour cycle were assessed [3]. The authors found that the endogenous melatonin rhythms of these crew members was on average 24.35 hours and concluded that despite social contact and the work environment they were not entrained by the 18-hour rest/activity cycle. A compressed-work schedule (ALT), designed to enhance circadian rhythm entrainment and sleep hygiene, was also tested on subjective sleep in 40 American submariners [4], with cortisol and temperature measurements in a sub-sample of 10 subjects. This schedule was reminiscent of the close 4 schedule designed by Kleitman and including 24 hours of watch-standing time per 72 hours at the non-nuclear time [5]. However sleep was found to be shorter with this new schedule and was therefore not retained as interesting by practioners compared to the classic 18 hour watch system [4]. Conversely, we found that the sleep quantity and quality of our subjects, exposed to a 24-h-3 days cycle did not change significantly during the 70-day mission. Subjectively, the submariners did not feel there was a significant difference in their quality of sleep at D21 and D51, and reported a relatively good quality of sleep and no mood alteration. Sleepiness, as assessed by the ESS, was slightly above normal and not significantly changed between the early and later stage of the mission. Objectively, we recorded normal amounts of sleep (around 400 minutes) at D21 and D51, with no significant change during the mission for TST, WASO, SOL, and the percentages of SWS and REM sleep [36]. REM latency and blood cortisol at the end of the night, which may be regarded as rough biological clock markers [37–39], were also not significantly changed. We can conclude that despite isolation and absence of natural light our sample of submariners had no significant objective or subjective sleep or wake disorders during a 70-day period. However, the TST and SE2 observed may be considered as relatively low, maybe even if not pathological. One may also argue that sleep may not be poorer at the end of the mission than it is at the beginning with a “floor effect”. This however did not fit with the subjective feeling of submariners, which describe their sleep as relatively good.

One strong point of our study, compared to the studies made before, was the possibility of observing submariners twice on D21 and D51 of this long-term mission with no influences by external potential cues. The previous studies carried out on 20 American submariners showed that an imposed 18-hour-cycle or compressed work schedules were not effective on synchronising rhythms [3–4]. Conversely we showed that the sleep quantity and quality of our subjects, exposed to a 24-h 3 days cycle was not significantly different on D21 and D51.

One possible explanation for the absence of sleep and wake complaints in our subjects despite shift work and the lack of stimulation by natural light lie in the particular work schedules of these submariners. Established by the Navy in the 17th century, the schedule allows a minimum of 8 hours of sleep per 24 hours, with a regular phase advance of 4 hours every day and organization of meals and rest synchronized to the shift "Fig 2". Social schedules onboard are perhaps enough to entrain the submariner to a 24 hour shift, in the absence of light, the main circadian synchroniser or “zeitgeber”. However, phase advance is generally not recommended in shift work organization and having to work every day is not usually found in industrial settings except in certain places, such as oceanic petroleum platforms or for astronauts [9,26]. Moreover, sleep disorders in shift workers are also a result of difficulties in combining work schedules and social life, and from constraints imposed by commuting times and disturbed environments at home for refreshing sleep [8,25–27,40]. The submariners have a full 8 hours devoted to sleep with no potential adverse effects of social or familial activities. We also acknowledged that when we recorded the second day of the shift "Fig 2" we maximized the opportunity of having a better sleep. Another possible reason for such good adaptation to their shift conditions is the absence of social and/or family obligations. The social isolation could thus potentially help the crew adapt to shift work [8,25–27,40]. The navy men’s social interaction may be a possible explanation for such good preservation. Communal meals at lunch and dinner were encouraged with their regular schedules between 11 and 12 a.m. and between 6 and 7 p.m. and due to living closely for such a long period may influence the men’s reciprocal rhythms. However these conditions were not sufficient enough to entrain American submariners on an 18-h-cycle [3]. Secondly, environmental issues such as lighting were specifically controlled, with low levels for security and power reasons, but adapted to the crew’s night and day rhythms, which may help improve sleep patterns [12,14–15]. Temperature and noise were also controlled "Fig 1". These factors may help submariners adapt more easily to shift schedules.

One could reasonably argue that submariners who are selected for the navy are in good physical shape, and are resistant to fatigue and night work; those with mental or severe chronic disorders are traditionally excluded from SSBN missions, but sleep disorders are not a cause for exclusion. We did not control the recruitment of the crew, nor did we select the volunteers via sleep criteria. Others may concur that the majority of these submariners were young males (12 between 19 and 29) with a good amount of SWS which may impact SE.

We do acknowledge some of the limitations of this study.

- Firstly, sleep patterns were only assessed twice during the mission and we did not obtain a baseline sleep assessment at the start of the mission. This decision was taken based on previous experience, which showed that the first few days of the mission are disturbed by the intensive workload before departure and difficulties linked to familial and social separation [30], such that recording sleep patterns at this time would not have been a true reflection of the crew members’ normal sleep pattern. We also understand that assessing sleep at D21 and D51 does not reflect the sleep patterns of the entire mission. Sleep was probably more difficult at the beginning due to shift-lag adaptation. However it is usually admitted that it is more difficult to maintain vigilance in the days in the middle of the mission than at the beginning or at the end [30–41].

- Secondly, we only recorded sleep patterns in 19 volunteer crew members and cannot confirm that this group was necessarily representative of the entire crew. It is possible that those who have better sleep patterns volunteered. However, it is traditional in the army to keep the first volunteers for a mission and for technical reasons it was not possible to study a larger group.

- Thirdly, REM latency and cortisol blood levels do not strictly reflect the adaptation of the biological clock; regular melatonin levels could have been a better means of assessing the clock’s adaptation [3,37–39]. However, performing a sleep study was not the priority of this SSBN mission and melatonin sampling (every hour for saliva samples in the evening) together with sleep recordings would have taken too much time for this type of security mission. We also understand that the time window for cortisol measurement was very large (12 hours) and did not allow estimating whether the clock was early or late. A morning void could have been more indicative.

- Finally, even if our data enabled us to observe that submariners did not significantly change their sleep between two periods of the mission we were not able to conclude that some of them were more adapted to those conditions than the others. The participants had served an average of 4.4 years on SSBN patrols and it could be suggested that no adapted subjects resigned, but we had no data on the general group of navy men that enabled us to conclude this point.

We believe that our findings may have important implications for considering new shifts of an increasing percentage of people working at night or shifts in continuous 24-hours industrialized societies, totally or incompletely isolated from natural light exposure, and in strategic missions for defence, communication, transportation, security and health. In these environments, workers may have to face acute and dangerous crisis, which demand a high level of alertness. In all these situations, as in our submariners, maintaining vigilance is crucial for our safety and peace. Any accidental collision of the submarine with the ground or with another ship would immediately jeopardize the crew’s survival. Good psychological and cognitive function is necessary to ensure the effectiveness of the mission in a SSBN submarine; maintaining good quality sleep has an important role to play in optimizing these elements.
